# A Systematic Review of Interventions to Change Staff Care Practices in Order to Improve Resident Outcomes in Nursing Homes

**DOI:** 10.1371/journal.pone.0140711

**Published:** 2015-11-11

**Authors:** Lee-Fay Low, Jennifer Fletcher, Belinda Goodenough, Yun-Hee Jeon, Christopher Etherton-Beer, Margaret MacAndrew, Elizabeth Beattie

**Affiliations:** 1 Faculty of Health Sciences, University of Sydney, New South Wales, Australia; 2 Dementia Collaborative Research Centre: Assessment and Better Care, University of New South Wales, New South Wales, Australia; 3 Dementia Study Training Centre, University of Wollongong, New South Wales, Australia; 4 Sydney Nursing School, University of Sydney, New South Wales, Australia; 5 School of Medicine and Pharmacology Royal Perth Hospital Unit, The University of Western Australia, Perth, Western Australia, Australia; 6 Dementia Collaborative Research Centre: Carers and Consumers, Faculty of Health, Queensland University of Technology, Brisbane, Queensland, Australia; University of Glasgow, UNITED KINGDOM

## Abstract

**Background:**

We systematically reviewed interventions that attempted to change staff practice to improve long-term care resident outcomes.

**Methods:**

Studies met criteria if they used a control group, included 6 or more nursing home units and quantitatively assessed staff behavior or resident outcomes. Intervention components were coded as including education material, training, audit and feedback, monitoring, champions, team meetings, policy or procedures and organizational restructure.

**Results:**

Sixty-three unique studies were broadly grouped according to clinical domain—oral health (3 studies), hygiene and infection control (3 studies), nutrition (2 studies), nursing home acquired pneumonia (2 studies), depression (2 studies) appropriate prescribing (7 studies), reduction of physical restraints (3 studies), management of behavioral and psychological symptoms of dementia (6 studies), falls reduction and prevention (11 studies), quality improvement (9 studies), philosophy of care (10 studies) and other (5 studies). No single intervention component, combination of, or increased number of components was associated with greater likelihood of positive outcomes. Studies with positive outcomes for residents also tended to change staff behavior, however changing staff behavior did not necessarily improve resident outcomes. Studies targeting specific care tasks (e.g. oral care, physical restraints) were more likely to produce positive outcomes than those requiring global practice changes (e.g. care philosophy). Studies using intervention theories were more likely to be successful. Program logic was rarely articulated, so it was often unclear whether there was a coherent connection between the intervention components and measured outcomes. Many studies reported barriers relating to staff (e.g. turnover, high workload, attitudes) or organizational factors (e.g. funding, resources, logistics).

**Conclusion:**

Changing staff practice in nursing homes is possible but complex. Interventionists should consider barriers and feasibility of program components to impact on each intended outcome.

## Introduction

There are multiple high quality trials and systematic reviews providing evidence for good practice in long-term residential institutions for older people, referred to in many countries as nursing homes and, also known as long-term care homes, homes for the aged, rest homes, residential aged care facilities [1–3]. However, there is often an unreasonable lag between research evidence and practice change [4]. Further, attempts at knowledge translation may not be successful. For instance, after over a decade of extensive promotion of person-centered cultures of care, culture change efforts are becoming widespread in American nursing homes, but it is not clear whether implementation efforts are changing staff and organizational practices, nor whether these practice changes are improving quality of care or resident outcomes [5].

Barriers to implementation have been identified such as cost, senior leadership resistance, low-innovation culture, low staff education, and high staff turnover [6]. Success factors for implementation include contextualizing the practice change, adequate resourcing, and demonstrating connections between practice change and outcomes [7].

Implementation science has an important role in bridging the gap between research and practice within health services [8]. There is a vast body of research that focuses on changing the practice of individual clinicians such as general practitioners [9,10], allied health professionals [11] and nurses [12]. There is less information about how to change the behavior of teams of staff in organizations such as hospitals, health services, and nursing homes, despite evidence suggesting that organizational culture contributes to health care performance [7,13].

Previous systematic reviews have examined whether specific interventions can improve related resident outcomes. For example, reviews have examined the effect of training nursing home staff in dementia care and management of behavioral and psychological symptoms, and the effectiveness of quality systems in improving nursing home quality of care and culture change [14–16] [17]. These reviews described the literature as being of relatively low quality with high possibility of methodological bias. The review of staff training concluded that extensive interventions with ongoing support successfully demonstrated practice change, but there was little evidence for simpler training without reinforcement [15]. The review of quality systems found that results were inconsistent but that there was some evidence that specific training and guidelines can influence resident outcomes [14]. These reviews focused on efficacy of interventions with less emphasis on identifying which interventions or components of interventions contributed to changing practice.

Implementation scientists are increasingly more interested in why practice change interventions succeed or fail and have called for greater use of theory in planning and understanding interventions [18]. Program logic models have also been used to describe how intervention components relate to each other and outcomes [19,20]. Articulating its logic to those delivering and receiving it may also help maintain its integrity during delivery [21].

This purposefully broad review aims to identify interventions or intervention components to change staff care practices in order to improve resident outcomes.

### Objectives

1. To systematically identify and describe studies that have investigated the effects of interventions to change staff practice or care approaches in order to improve resident outcomes in nursing homes;
2. To identify interventions or intervention components which lead to successful staff practice or care approach change in nursing homes;
3. To identify potential barriers and enablers to staff practice or care approach change in nursing homes.

## Methods

### Literature search

The search strategy was developed following consultation with an information services university librarian using an iterative process of preliminary searches testing search terms and incorporating new search terms as relevant papers were identified. In addition to our own search terms, our strategy included all relevant MeSH (Medical Subject Heading) terms. Using language (English) and date (1990–5th December, 2013) restrictions and searching titles, keywords and abstracts, we systematically searched the following electronic databases: Ovid MEDLINE, PubMED (from 2012 onwards as up to 2012 would be covered in MEDLINE), Scopus (Health sciences and social sciences), Cochrane Central Register of Controlled Trials, Cochrane Database of Systematic Reviews, CINAHL (Cumulative Index to Nursing and Allied Health Literature), PsycINFO, and Database of Abstracts of Reviews of Effects. Reference lists of included papers and related reviews were hand searched. The “grey literature” was not specifically searched. Search results were combined using the electronic referencing system Endnote, and duplicate citations were removed.

General search strategy: (“nursing home?” or “long?term care” or “residential care” or “home? for the aged” or “residential facilit*” or “residential aged care”) And (“implementation” or “knowledge translation” or “knowledge transfer*” or “culture change” or “adoption” or “quality improvement” or “dissemination” or “diffusion” or “practice change” or “training” or “champion?” or “opinion leader?” or “educational outreach” or “case conference” or “audit and feedback” or “organisational change” or “organizational change” or “”professional development” or “supervision” or “leadership” or “health plan implementation” or “traditional medical research” or “organi?ational culture” or “organi?ational innovation”) And (“staff” or “carer?” or “management” or “nurse?” or “careworker?” or “manager?” or “personal support worker?” or “personnel” or “caregivers” or “health personnel”).

### Study selection

Two researchers (LFL and JF) independently screened the titles and abstracts and determined whether a study met inclusion criteria. The full text of all articles classified as meeting or possibly meeting inclusion criteria were retrieved and evaluated. Disagreements were resolved by discussion between the two reviewers.

### Inclusion criteria

#### Setting

Studies were conducted in nursing homes, i.e. facilities catering for permanent residential care of older people including providing housekeeping, personal care, meals, activities and nursing home. This is distinct from medical facilities primarily delivering medical or palliative treatments, and retirement villages where residents attend to their own personal care and housekeeping.

#### Study design

Randomized controlled trials and quasi-experimental controlled trials were included as recommended by the Cochrane Effective Practice and Organisation of Care (EPOC) group [22].

#### Sample size

Only studies with 3 or more sites in each group were included. EPOC recommends only including clustered trials with at least two intervention sites and two control sites. The rationale was that in studies with only one intervention or one control site, the intervention is completely confounded by site characteristics making it difficult to attribute any observed differences to the intervention rather than to other site-specific variables. We extended this requirement to at least three intervention and three control sites in order to reduce the possibility of site-specific confounding and increase generalisability. A study with fewer than 6 sites is unlikely to be statistically powered to take into account site clustering in the analysis. Studies were not restricted based on the number of participants within each site.

#### Interventions

Aimed at changing the care practices of staff for the benefit of the residents. The intervention or components of the intervention were not delivered directly to residents by the research team or other external clinicians.

#### Outcome measures

Empirically assessed change in at least one of the following outcomes: change in staff behavior (but not just attitudes or knowledge), change in other staff outcomes (e.g. staff turnover, absenteeism or stress) change in resident clinical outcomes (but not just satisfaction with care). We did not include studies in which the only outcomes were staff attitudes or knowledge as changing knowledge does not necessitate change in behavior [23,24], or those in which the only resident outcome was satisfaction with care as these represent overly an optimistic view of care [25].

### Data extraction

Study data were extracted using standard forms that were based on forms developed by the Cochrane Effective Practice and Organisation of Care Group [22]. Extraction was conducted by one researcher (LFL or JF) and checked by a second researcher (JF, LFL or MM). Study authors were contacted for additional information as required.

#### Categorising of intervention components

We categorized interventions via their different components (one intervention could have many components) according to categories and definitions adapted from the Cochrane EPOC group [22]. These were:

- Educational material: written material or a DVD/video or online website
- Training: delivered in person to staff
- Reminders: e.g. postcards, posters—designed to prompt practice
- Audit and feedback: formal monitoring of the performance of staff or the organization which is fed back to them
- Mentoring or support: supervision/consultation/mentoring of staff in teams or individually to support practice change
- Champions: individuals or teams responsible for driving change within the site
- Team meetings: Consensus/multidisciplinary team meetings to discuss issues relating to the clinical domain of practice
- Policy/procedure: a new policy or procedure introduced into the organization (e.g. reporting tool, assessment tool, guideline)
- Organizational restructure: change to the responsibilities of staff or the way care is organized

#### Barriers and enablers

Information on barriers and enablers were extracted either where reported as part of a process evaluation, or as part of the discussion section.

#### Theoretical models of behavior change

We collected information on theoretical models described as underpinning behavior change strategies. These were differentiated from theoretical models guiding the hypothesized relationship between the intervention and the resident outcomes.

#### Program logic models

Program logic models attempt to identify key program components and outcomes and depict how these elements are expected to relate to each other [26]. Program logic models help researchers identify weaknesses in hypothesized causal relationships between intervention components and desired outcomes. Program logic models are also useful in planning evaluations [20].

**Fig 1:**
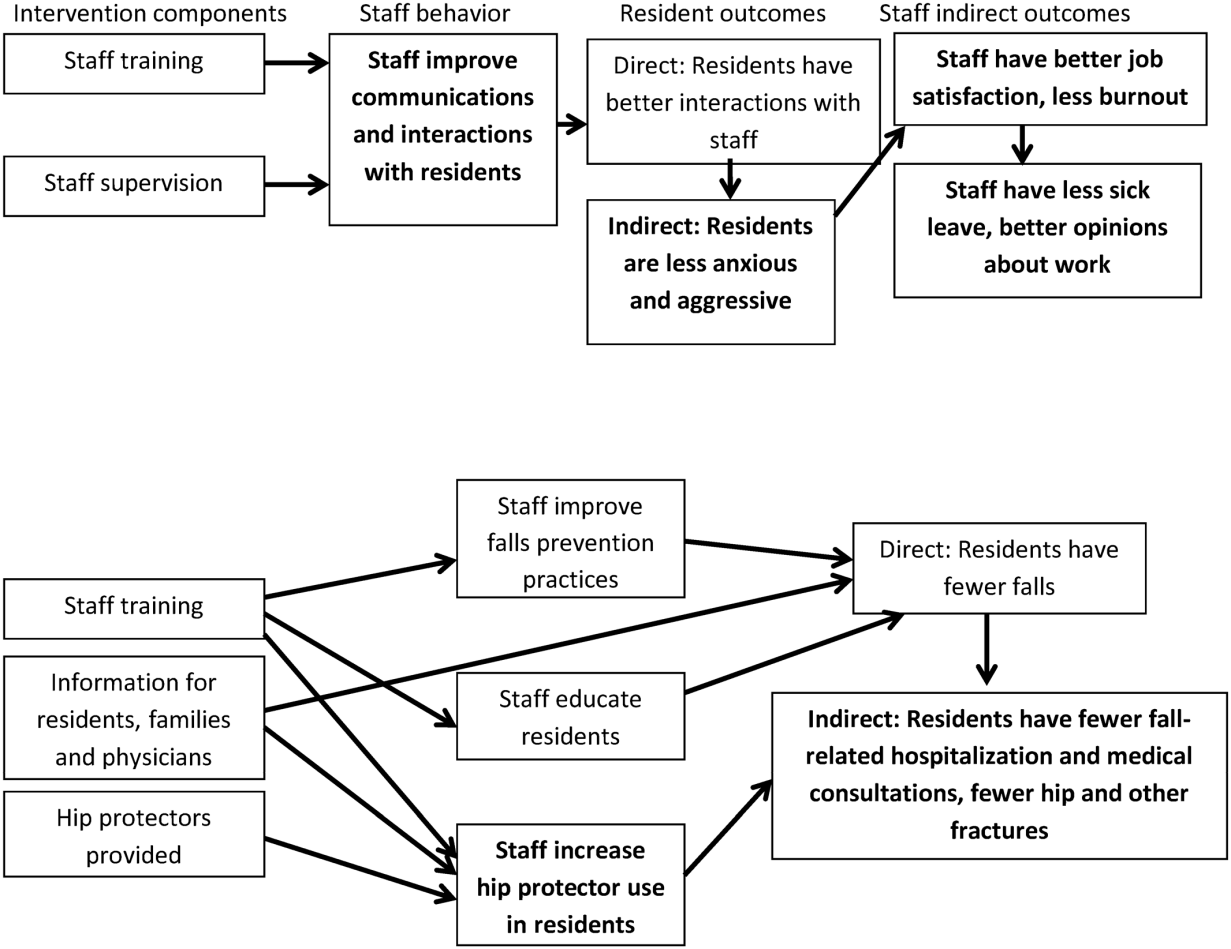
Program logic drawn for Schrijnemaekers et al (2002) and Meyer (2005). Measured outcomes shown in **bold**.

Where the program logic was described in a figure or text, this was extracted. Otherwise researchers drew a program logic model based on their interpretation of the description of the study (see examples in Fig 1). We used the program logic model to help us categorize outcomes into staff behavior (behavior that is directly targeted by the intervention), staff indirect outcomes (staff characteristics and behaviors not directly targeted by the intervention such as turnover and stress) and resident outcomes (both directly targeted and indirectly assumed to be impacted by the intervention). We examined these models for weaknesses in the relationships between the intervention and outcomes, as well as staff behaviour changes, or resident outcomes that were implicit but not measured in the evaluation.

#### Risk of bias

One reviewer assessed the risk of bias of included studies as outlined in the Cochrane Risk of Bias for EPOC reviews tool [22] that considers selection bias, performance bias, detection bias, attrition bias, reporting bias and other bias. This risk assessment was checked by a second reviewer and disagreements were resolved through discussion.

### Data analysis and synthesis

The purpose of this review was not to evaluate the efficacy of interventions. Within each clinical domains there were no studies with similar intervention components and outcome measures which could be considered for combination in meta-analysis. Hence meta-analyses were not undertaken. Results are presented in narrative form.

Studies were according to the clinical domains in which practice change was targeted. Clinical domains were then ordered according to our subjective judgement of the complexity and difficulty of the behavior change required and are presented in that order from Tables 1 to 12. There is no model or framework for classifying how complex or difficult a behaviour is to change, particularly in an organisational context, however this is intuitively an important factor to consider in this review. In ranking domains by difficulty of behavior change, we considered whether there were salient cues for the new behaviors with cues making change easier, whether past habitual behavior had to be relearnt as this is more difficult than learning a new behavior, whether the practice change required coordination and cooperation between multiple staff members which we ranked as more difficult than when cooperation was not required, and the frequency in which the behaviors occur where more frequent behaviors were harder to change [27].

**Table 1:** Oral Health.

First author, year	Study design, intervention length, follow-up, number of sites, baseline sample size	Control condition	Education material	Training	Reminders	Audit and feedback	Mentoring or support	Champion/s	Team meetings	Policy/procedure changes	Organizational restructure	Intervention description	Staff direct behavior outcomes	Staff indirect outcomes	Resident outcomes
Frenkel, 2001	Block cluster randomized RCT, 1 wk, 26 wk follow-up, 22 sites (11 each), CG: 211 res; IG: 201 res	Usual care		+								Training for staff in oral health care, role of plaque in oral disease, cleaning techniques for dentures and natural teeth. Toothbrushes were distributed to all clients			Compared to CG, over time the IG improved on denture and dental plaque, gingivitis, denture induced stomatitis and the proportion of residents with erythema or papillary hyperplasia. There were no differences between groups over time for calculus, root caries and tooth mobility.
De Visschere, 2012	Stratified, cluster randomized RCT, 26 wks, 12 sites, CG: 186 res; IG: 187 res	Guidelines only		+			+	+				Manager appointed as a project supervisor to lead oral health care team—including nursing staff, physician, occupational or speech therapist. Presentations on guidelines, daily oral health care protocol and supervised implementation project. Training for oral health team who then trained all nursing staff. Oral health team had to encourage and assist staff in daily delivery of oral health care. Free oral health care products supplied. Monitoring visits by research staff.			Compared to CG, over time the IG had a small improvement in denture plaque. No significant intervention effects for dental plaque and tongue plaque.
Van der Putten, 2013	Stratified cluster randomized RCT, 26 wks, 12 sites, CG: 165 res; IG: 177 res	Guidelines only		+			+	+				Same intervention as above (conducted in different countries).			Compared to the CG, IG improved in dental plaque and in denture plaque.

RCT, randomized controlled trial; CG, control group; IG, intervention group; wks, weeks; res, residents.

**Table 2:** Hygiene and infection control.

First author, year	Study design, intervention length, number of sites, baseline sample size	Control condition	Education material	Training	Reminders	Audit and feedback	Mentoring or support	Champion/s	Team meetings	Policy/procedure changes	Organizational restructure	Intervention description	Staff direct behavior outcomes	Staff indirect outcomes	Resident outcomes
Makris, 2000	Paired, clustered randomized RCT, 52 wks, 8 sites, CG: 447 res; IG: 443 res	Usual policy	+	+		+						Training on infection, preventing disease transmission, control of food borne illness, food safety, cleaning and disinfecting surfaces and equipment. Handouts provided. Infection control nurse and departmental directors of homes responsible for providing education to new staff. Certified infection control professionals provided on-site visits. Germicidal products provided.			No differences between groups on combined infections, genitourinary, cutaneous, lower respiratory, gastrointestinal, other or upper respiratory infections.
Baldwin, 2010	Paired, randomized RCT, 52 wks, 32 sites, CG: 169 staff, 401 res; IG: 164 staff, 392 res	Usual care		+		+		+				Training for all staff—hand hygiene and decontamination demonstrations, audit and feedback of infection control, link worker to reinforce good infection control.	Compared to CG, IG infection control audit scores were higher over time.	No difference between groups in staff methicillin resistant staphylococcus aureus (MRSA)	No differences between groups on residents with methicillin resistant staphylococcus aureus (MRSA)
Ho, 2012	Cluster randomized RCT, 4 wks, 4 months follow-up, 18 sites, CG: 711 res, 231 staff; IG1: 767 res, 248 staff;	Usual care	+	+	+	+	+					IG1: Slightly powdered gloves provided at points of care. Posters and reminders. Training on hand hygiene. Observations of hygiene in practice with feedback offered.	Compared to CG, IG1 and IG2 both showed improved hand hygiene.		Compared to CG, both IGs showed reduction in hospitalization related to respiratory outbreaks or methicillin resistant staphylococcus aureus (MRSA) infections.
	IG2: 929 res, 331 staff			+	+		+					IG2: As for IG1 except gloves were powderless and no specific feedback.			

RCT, randomized controlled trial; CG, control group; IG, intervention group; IG1, intervention group 1; IG2. Intervention group 2; wks, weeks; res, residents.

**Table 3:** Nutrition.

First author, year	Study design, intervention length, number of sites, baseline sample size	Control condition	Education material	Training	Reminders	Audit and feedback	Mentoring or support	Champion/s	Team meetings	Policy/procedure changes	Organizational restructure	Intervention description	Staff direct behavior outcomes	Staff indirect outcomes	Resident outcomes
Westergren, 2009; 2010	NRCT, variable duration, 65 sites, CG: 1084 res; IG1: 175 res;	Usual care	+		+		+		+		+	IG1 = policy document.	IG1 had some short and long term positive effects and IG2 had some positive short term effects on nutritional care.		No group by time differences between IG1, IG2 or CG on undernutrition risk and overweight. No change in the number of residents with low or high BMI in the CG, but a significant decrease in people with low BMI in IG2. There was a significant increase in residents with high BMI in the IG1.
	IG2: 467 res		+	+	+			+	+			IG2 = study circles. Study circles including kitchen and nursing staff met. Circles created a structured change plan. Study circle leader trained.			
Gaskill, 2009	Cluster randomized RCT, 26 wks, 8 sites, IG: 134 res; CG: 145 res	posters	+	+			+	+				Train-the-trainer. Researchers trained nutrition coordinators for each facility; nutrition coordinators trained facility staff using supplied materials and were responsible with liaising with nursing, kitchen and domestic staff, and facilitating in-service sessions about the nutrition strategies.	Compared to CG, IG was more likely to receive high energy, high protein diet and less likely to have pureed meals or thickened fluids or require assistance feeding. No differences between groups over time on consultations with dietician or speech pathologist.		Compared to CG, IG were more likely to maintain or improve their nourishment rating. No significant differences on risk of being malnourished.

NRCT, non-randomized controlled trial; RCT, randomized controlled trial; CG, control group; IG, intervention group; IG1, intervention group 1; IG2. Intervention group 2; wks, weeks; res, residents; BMI, Body Mass Index.

**Table 4:** Nursing home acquired pneumonia (NHAP) prevention and management.

First author, year	Study design, intervention length, number of sites, baseline sample size	Control condition	Education material	Training	Reminders	Audit and feedback	Mentoring or support	Champion/s	Team meetings	Policy/procedure changes	Organizational restructure	Intervention description	Staff direct behavior outcomes	Staff indirect outcomes	Resident outcomes
Hutt, 2010, 2011; Linnebur, 2011	NRCT, 104 wks, 16 sites, CG: 549 res; IG: 574 res	Usual care	+	+	+			+				$2000 per annum payment per home, meetings with DON and administrator of each facility to present NHAP guidelines, and discuss barriers. Nurse championed intervention and encouraged vaccination for staff and residents. Staff training on vaccination, vaccinations available at in-services.	No differences in guideline adherence for treating residents with NHAP over intervention. Compared to CG, greater improvement in IG direct care staff influenza vaccination and resident pneumococcal vaccination rates. No differences in resident influenza vaccination rates.		No differences in hospitalization rates or mortality rates over intervention period. No difference in mortality rate over 3 years.
Naughton, 2001	Cluster randomized RCT, 1 wk, 52 wks follow-up, 10 sites, 350 episodes of NHAP across groups	Pneumonia guidelines presented to physicians only	+	+	+				+			Nursing home acquired pneumonia guidelines presented to physicians and nurse practitioners. Staff trained. Nursing staff prompted to identify barriers to implementation and develop strategies for dealing with them. Laminated pocket cards for all RNs and LPNs.	No differences between IG and CG—both increased on parenteral antibiotics but not oral antibiotic use.		Indirect outcomes:- Compared to CG, IG had no differences in hospitalization or 30-day mortality.

NRCT, non-randomized controlled trial; RCT, randomized controlled trial; CG, control group; IG, intervention group; IG1, intervention group 1; IG2. Intervention group 2; wks, weeks; res, residents; NHAP, nursing home acquired pneumonia, RNs, registered nurses; LPNs, licensed practical nurses.

**Table 5:** Depression.

First author, year	Study design, intervention length, number of sites, baseline sample size	Control condition	Education material	Training	Reminders	Audit and feedback	Mentoring or support	Champion/s	Team meetings	Policy/procedure changes	Organizational restructure	Intervention description	Staff direct behavior outcomes	Staff indirect outcomes	Resident outcomes
Leontjevas, 2013	Stepped wedge cluster randomized RCT, 26 wks, follow-up varied 0–16 months, 33 sites, CG and IG number varied	Usual care	+	+						+		Multi-disciplinary care program that prescribed pathways for collaborative treatment: structured assessment, 2 step screening and diagnostic procedure. Multidisciplinary treatment. Monitoring of treatment effects. Information and practical tools were provided, staff trained on depression and the program, psychologists trained on life-review therapy training, medication protocol to the unit physician, tailored communication with psychologists and physicians about individual depression scores.			Dementia and somatic units’ results treated separately. No intervention effects on depression in dementia units, but clinical depression decreased (on the main measure) after crossing to the intervention in somatic units. Indirect outcomes: quality of life improved after intervention in both somatic and dementia units.
Smith, 2013	NRCT, 6 wks, 8, 12 and 16 wks post-training follow-ups, 13 sites, CG: 13 res; IG1: 16 res; IG2: 30 res	Usual care	+	+								IG1: CD-based training (self-directed) on late-life depression and comorbid conditions, rating scales, interventions (psychosocial, behavioral activation, medication use), communication and teamwork among health providers. Learners implemented exercises with an older adult with depression			IG results reported together, not individually. No group by time effects on depression. Indirect outcomes: No differences between groups over time for anxiety or quality of life or pain.
			+	+			+					IG2: CD-based training with psychiatric nurse support involving weekly phone calls, on-site visits.			

NRCT, non-randomized controlled trial; RCT, randomized controlled trial; CG, control group; IG, intervention group; IG1, intervention group 1; IG2. Intervention group 2; wks, weeks; res, residents.

**Table 6:** Appropriate prescribing.

First author, year	Study design, intervention length, number of sites, baseline sample size	Control condition	Education material	Training	Reminders	Audit and feedback	Mentoring or support	Champion/s	Team meetings	Policy/procedure changes	Organizational restructure	Intervention description	Staff direct behavior outcomes	Staff indirect outcomes	Resident outcomes
Avorn, 1992	Paired, clustered randomized RCT, 22 wks, 12 sites, CG: 392 res; IG: 431 res	Usual care	+	+			+					Education material sent to physicians followed by educational visits with each physician with high psychoactive drug prescribing rate. Training for all direct care and nursing staff.	Compared to CG, in IG there was greater reduction of mean psychoactive drug use and number of days of antipsychotic therapy per month, and more discontinuation of antipsychotics		Indirect outcomes: compared to CG, IG residents on baseline antipsychotics had greater maintenance of memory but worsened depressive symptoms. No differences on mental state, anxiety, other behavior or sleep. Compared to CG, IG residents on baseline benzodiazepines or hypnotics decreased in anxiety and improved in function, deteriorated more in memory. No differences between groups in rates of hospitalization, mortality or change in level of care.
Meador, 1997	Paired, clustered randomized RCT, 5 wks, 5 month follow-up, 12 sites. CG: 631 res; IG: 680 res.	Usual care	+	+	+			+	+			Physician visited, given prescription recommendations and flowchart. Staff trained in structured guidelines: medical evaluation; minimizing the occurrence and severity of behavioral problems; written plans to manage behavioral problems; low dose antipsychotic therapy for behaviors that were dangerous, interfered with care or seriously distressed the resident; trials of gradual withdrawal	Reduced use of antipsychotics in intervention compared to control; no increase in benzodiazepine use.		Reduced use of antipsychotics in intervention compared to control; no increase in benzodiazepine use.
Schmidt, 1998	Paired, clustered RCT, 52 wks, 33 sites, CG: 1228 res; IG: 626 res	Usual care		+			+		+			Trained pharmacists spent 1 day outreach per month at each home. Pharmacists organized regular multi-disciplinary drug use meetings for staff involved in drug administration and/or direct resident care.	In CGs, there were significant increases in number of drugs prescribed and proportion of residents with therapeutic duplication; these were stable in IG. In IG, use of recommended hypnotics increased and non-recommended hypnotics decreased and overall rate declined, no significant changes in CG. No change in either group in use of non-recommended anxiolytics. Increase in IG in the promotion of residents with acceptable anxiolytics. Both groups decreased in the use of tricyclic antidepressants (non-preferred treatment) and both groups increased in the use of selective serotonin re-uptake inhibitors (a preferred treatment).		
Crotty, 2004	Cluster randomized RCT, 12 wks, 10 sites, CG: 104 res; IG: 50 res.	½ day training and a toolkit on management of challenging behavior	+	+					+			Medical outreach team trained on toolkit in management of challenging behaviors. A problem list and medication review was conducted by GPs and care staff and presented at 2 case conferences which also included a geriatrician, pharmacist, and representative of the Alzheimer’s Association.	Compared to the CG, the change scores for medication appropriateness index (MAI) improved. Compared to the CG, the MAI scores for benzodiazepines (considered inappropriate) were significantly reduced.		No differences between groups on behavior. No carry-over effects to other residents in the facility.
Fossey, 2006	Stratified block cluster randomized RCT, 42 wks, follow-up at 52 wks, 12 sites, CG: 168 res; IG: 181 res	Psychiatric drug review, letters and phone calls to GPs		+			+					Nursing staff trained in person centered care, positive care planning, environmental design, individualized interventions, alternatives to drugs, involving family carers. Group and individual supervision including addressing organizational change. Physician consulted about recommendations	Compared to CG, proportion of IG participants taking neuroleptics was significantly lower.		No differences between groups for agitation. Indirect outcomes: no differences between groups on falls or wellbeing.
Westbury, 2010, 2011	NRCT, 26 wks, 78 wks follow-up, 25 sites, CG: 693 res; IG: 898 res	Usual care	+	+	+	+			+	+		Drug use evaluation data provided. Staff training. Individualized academic detailing for physicians. Educational pamphlet for residents and relatives. “Sedative review” form generated—to be discussed by staff, GP, carer and pharmacist.	Over the 6 months intervention, compared to CG, IG had reduced use and dose of benzodiazepines, antipsychotics, overall psychotropic use, and multiple psychotropic use. No differences between groups on antidepressant use. At follow-up: Decrease in benzodiazepines and diazepam use and dosage carried out to 18 months but the prescription and dosages of antipsychotics returned to baseline in IG homes at 18 months.		
Stein, 2001	Paired, clustered, RCT, 13 wks, 20 sites, CG: 71 res; IG: 76 res	Usual care	+	+						+		Trainers in an educational program for optimal treatment of musculoskeletal pain for all nursing home staff: alternative approaches to NSAIDS. Meeting with administrator and DON. Meeting with the nursing home appointed study coordinator. Visited or telephoned all primary care physicians of participants in intervention homes. Algorithm for stopping NSAIDS in high risk persons. Educational materials for physicians and appointed nurse coordinator.	Effectively decreased non-steroidal anti-inflammatory drugs (NSAIDs) use and increased acetaminophen use. Mean number of days of NSAID use dropped over 3 months and increased for acetaminophen.		Pain did not change over time. No measure of functionality was significantly different. There were no significantly different mean changes from baseline to follow-up between IG and CG in illness impacts, cognition, gastrointestinal symptoms or difficulty with activities of daily living.

NRCT, non-randomized controlled trial; RCT, randomized controlled trial; CG, control group; IG, intervention group; IG1, intervention group 1; IG2. Intervention group 2; wks, weeks; res, residents; GPs, general practitioners; NSAIDS, non-steroidal anti-inflammatory drugs; DON, Director of nursing.

**Table 7:** Physical restraint use.

First author, year	Study design, intervention length, number of sites, baseline sample size	Control condition	Education material	Training	Reminders	Audit and feedback	Mentoring or support	Champion/s	Team meetings	Policy/procedure changes	Organizational restructure	Intervention description	Staff direct behavior outcomes	Staff indirect outcomes	Resident outcomes
Huizing, 2009	RCT, 26 wks, 15 wards in 7 nursing homes, CG: 163 res; IG: 208 res	Usual care		+			+					Training for all staff on physical restraints—effectiveness, consequences, decision making processes, strategies for analyzing and responding to residents’ risk behavior. Extensive training for nursing staff with key roles on ward. Consultant visited weekly, advised nursing staff, attended multidisciplinary meetings, evaluated the use of physical restraints and discussed difficulties.	In both groups use of restraints and intensity of restraints increased over time. Compared to the CG, over time IG had decreased use of sleep suits, use of belts in bed, bilateral bedrails, and increased use of deep/tipped chairs, increased use of belts and increased use of infrared systems.		
Gulpers, 2011, 2013	NRCT, 35 wks, 13 nursing homes, 26 wards, CG: 201 res; IG: 317 res	Usual care; control received treatment after 35 wks		+			+			+		Policy change by management prohibiting new use of belts and for reduction of current use. Education for staff. Consultation to ward nurses by nurse specialists regarding challenges in reducing restraints and specific resident issues. Provision of alternative interventions e.g. sensor mats, balance training, exercises, low—height adjustable beds.	Compared to CG, IG significantly decreased over time on belt use and on any type of physical restraints. No differences between groups over time in psychotropic use. Decrease in restraint use continued through to 24 months.		Indirect outcomes: No differences between groups on falls or injuries.
Kopke, 2012	Stratified, block randomized RCT, 26 wks, 36 sites, CG: 1819 res; IG: 1952 res	Print information and short presentation	+	+				+				Training on guidelines on physical restraints and alternative approaches. Guidance provided on posters, pens, mugs, and notepads. Nominated nurse from each cluster home trained on implementation process. Endorsement by nursing home leaders.	Compared to CG, IG reduced in prevalence of physical restraints. No differences between groups in psychotropic use.		Indirect outcomes: no differences between groups on falls or fall-related fractures.

NRCT, non-randomized controlled trial; RCT, randomized controlled trial; CG, control group; IG, intervention group; wks, weeks; res, residents.

**Table 8:** Management of behavioral and psychological symptoms of dementia.

First author, year	Study design, intervention length, number of sites, baseline sample size	Control condition	Education material	Training	Reminders	Audit and feedback	Mentoring or support	Champion/s	Team meetings	Policy/procedure changes	Organizational restructure	Intervention description	Staff direct behavior outcomes	Staff indirect outcomes	Resident outcomes
Proctor, 1998, 1999	Paired, clustered RCT, 26 wks, 12 sites, CG: 60 res, IG: 60 res	Usual care		+			+					Training on organic and functional disorders in old age, approaches to care, activities. Supervision in individual program planning including observation and assessment, perceived needs and goal planning, breaking down the goal into steps and small targets, monitoring and recording progress.		At follow-up a larger proportion of CG staff met criteria for caseness on the General Health Questionnaire compared with IG staff. No differences over time between groups on sources of work related pressure.	Compared to CG, over time IG had less cognitive decline and more improvement in depression. No differences between groups over time on level of behavior or physical disability.
Deudon, 2009	Cluster, randomized RCT, 8 wks, follow-up at 12 wks, 16 sites, CG: 132 res; IG: 174 res	Usual care	+	+	+	+	+					Training on behavioral and psychological symptoms of dementia (BPSD), “how to” staff instruction cards on managing BPSD, what to do and what to avoid in care, non-pharmacological interventions. Personalized staff consultation.			Compared to CG, over time the IG had significantly lower global agitation, physically non-aggressive, verbally non-aggressive behaviors, and observed behavioral disturbances.
Zimmerman, 2010	Nested cohort RCT, 6 wks, 3 months follow-up, 16 sites, CG: 371 staff; IG: 291 staff	Usual care		+								Training for supervisors and direct care staff on dementia care and pain reduction. Supervisors trained on leadership skills.	Immediate improvement in communication and in pain awareness and after 3 months, communication improvements persisted.	Compared to the CG, the IG work stress increased and supervisory support received decreased for direct care staff and supervisors.	
Van de ven, 2013	Minimization on cluster randomized RCT, 35 wks, 34 sites, CG: 198 staff, 166 res; IG: 178 staff, 102 res			+								2 staff members trained in Dementia Care Mapping (DCM). DCM briefing day for organization.		Compared to CG, over time the IG reported fewer negative emotional reactions and more positive emotional reactions, greater autonomy and work pleasure. No differences between groups on general health or job satisfaction.	Compared to CG, IG deteriorated on total neuropsychiatric symptoms. No differences between groups over time on agitation. Indirect outcomes: no differences between groups on quality of life.
Leone, 2013	Cluster, randomized RCT, 4 wks, 13 wks follow-up, 16 sites, CG: 111 res; IG: 119 res			+	+							Training for staff on apathy in dementia, depression, deficits in function, structured activities; information including recommendations for non-pharmacological interventions summarized on Dos and Don’ts card.		No differences between groups on prescriptions of psychotropics, antidepressants, anxiolytics or antipsychotics.	Compared to CG, IG improved on emotional blunting, but not an initiative or interest, and had greater deterioration on affective and psychotic symptoms. IG had improvements on some activities of daily living (dressing and transferring), and deterioration on others (toileting and continence).
Eisses, 2005	Paired, cluster randomized RCT, 2 wks, 26 wks follow-up, 10 sites, CG: 228 res; IG: 198 res			+			+			+		Training for staff on differences between dementia and depression behaviors. Video material. Use of standardized screening instrument for depression, reviews at staff meetings.	Improvement in sensitivity, but not specificity, in recognition of depression symptoms. No improvements in treatment of depression.		Improvement in depressive symptoms. No difference for prevalence and incidence of depression.

NRCT, non-randomized controlled trial; RCT, randomized controlled trial; CG, control group; IG, intervention group; wks, weeks; res, residents.

**Table 9:** Falls reduction and prevention.

First author, year	Study design, intervention length, number of sites, baseline sample size	Control condition	Education material	Training	Reminders	Audit and feedback	Mentoring or support	Champion/s	Team meetings	Policy/procedure changes	Organizational restructure	Intervention description	Staff direct behavior outcomes	Staff indirect outcomes	Resident outcomes
Meyer, 2003, 2005	Cluster, randomized RCT, 78 wks, 49 sites, CG: 483 res; IG: 459 res	Information on falls	+	+								Training for staff on risk of hip fracture and related morbidity, strategies to prevent falls and fractures, effectiveness of hip protectors, protector use and implementation. Staff asked to educate residents. Provided hip protectors, flip charts and leaflets for residents, information for relatives and physicians.	Compared to CG, greater proportion of IG residents used hip protectors.		Compared to CG, IG had fewer hospital admissions related to falls. No differences between groups in risk of having one fall, mean number of falls or risk of hip fractures or other fractures, or in number of falls-related medical consultations.
O’Halloran, 2004	Stratified block randomized RCT, 72 wks, 127 sites, CG: 2761 res; IG: 1366 res	Usual care	+	+	+		+			+		Support obtained from and protocol provided to managers and organizations. Support to homes to promote and monitor progress. Training on use of hip protectors, risks and consequences of fractures. Reminder posters and stickers. Video provided to be used by staff. Information sessions for residents and families made available. Four pairs of hip protectors provided for every resident agreeing to wear them.	Initial acceptance of hip protectors was 37.2% in the intervention group with adherence falling to 19.9% at 72 weeks.		Compared to CG, IG increased in pelvic fracture rate and no difference in overall mean fracture rate or injurious falls.
Kerse, 2004	Stratified block randomized RCT, 26 wks, follow-up at 52 wks, 14 sites, CG: 309 res; IG: 238 res	Usual care		+		+		+		+		Falls coordinator appointed and trained. Training for staff on falls risk assessment and management, logo to identify high risk residents, color-coding for residents’ plans. Falls prevention manual containing risk assessment forms, fall prevention strategies, logos provided. Audit and feedback of falls.			Compared to CG, IG had a higher incidence rate of falls. There were no differences between groups on injurious fall incidence or serious injuries.
Ray, 2005	Stratified block cluster randomized RCT, 1 wk, follow-up at 52 wks, 112 sites, CG: 5626 res; IG: 4932 res	Usual care	+	+	+		+					Program teams trained comprising: nurse to select, assess and develop care plans for residents at high risk for falls, monitor staff performance and conduct in-service training; nursing assistants to inspect resident living space and equipment; occupational and physical therapy assistants to assess transferring and mobility to recommend wheelchair seating modifications; engineer to inspect and repair wheelchairs. Manual, video, materials for staff in-service, assessments and to track treatment plan implementation provided. Regular phone support.			No differences between groups on hip fractures, other fractures, soft tissue injuries or total injuries.
Wagner, 2005	NRCT, 17 wks, 6 sites, 910 res	Usual care	+			+				+		Falls menu-driven incident-reporting system (MDIRS) replaced existing narrative reporting. Training manual provided.	Compared to CG, in IG higher proportions documented of near falls, type of footwear, fall types, circumstances and side rail status. No differences between groups for documentation of unknown fall outcomes or pain.		No differences between groups on incidence of falls.
Rask, 2007	NRCT, 52 wks, 42 sites, CG: 19 sites; IG: 23 sites	Usual care	+	+			+	+		+		Organizational support developed and facility prepared. Falls team trained and quality improvement tools provided. Falls nurse coordinator appointed to champion the program. Support given during implementation.	Reduction in physical restraint use in both CG and IG. IG had improvements across time in care process documentation associated with falls management.		IG did not change in fall rate, but CG increased. No significant changes in serious injuries resulting from falls in CG or IG.
Cox, 2008	Stratified, cluster randomized RCT, 52 wks, 230 sites, CG: 2753 res; IG: 3476 res	Usual care, delayed treatment		+			+			+		Managers, RN and NAs trained on fall risk assessment and fracture prevention. Staff assessed residents, calculated fall and fracture risk, reported results to care home and GP with recommendation	Compared to CG, in IG there was a significant increase in bisphosphonate, calcium, and Vitamin D prescriptions. No differences in hip protector use.		No differences between groups for falls, total fractures or hip fractures.
Bouwen, 2008	Paired (and some unpaired) cluster randomized RCT, 6 wks, 26 wks follow-up, 10 sites, CG: 169 res; IG: 210 res	Usual care	+	+	+							Training for staff on risk factors for falls and environmental and behavioral modifications. Reinforcement with reminders. Nurses kept diary of falls to list risk factors and possible preventive interventions. Information collected on medications and comorbidities for residents with risk factors for falls.			Compared to CG, in IG there was a significant reduction of residents with one or more falls. For residents with 1+ fall, no difference in average number of falls between groups.
Rapp, 2010	NRCT, 52 wks, 104 wks follow-up, 1359 sites, CG1: 23,250 res; CG2: 20,333 res; IG: 9,077 res	Usual care, CG1: from within same state; CG2: from other state		+		+		+				Audit and feedback regarding a number of falls. Manual provided. Training for physiotherapists/ exercise instructors in conducting exercise groups. Weekly group exercise programs. Fall prevention nurses trained who were responsible for in-house teaching sessions and implementation. Web-training materials provided. Medication review for residents focusing on reducing psychotropics and administration of vitamin D. Environmental hazards identified. Hip protectors recommended. Fall risk tool used. Regular support visits.			No significant differences between CGs and IG on incidence of femoral fractures.
Becker, 2011	NRCT, 52 wks, 1149 sites, CG: 31,668 res; IG: 13,653 res	Usual care	+	+		+		+	+			Homes signed participation contract. Training for change agents and exercise instructors. Manual provided and materials for in-house education, web page with additional information. Group progressive strength and balance training exercises delivered biweekly by exercise instructors and staff. Documentation of falls. Environmental checklist to identify person-environment mismatch. Medication review and Vitamin D discussed with physicians. Hip protectors provided for demonstration and recommendations.			Compared to CG, IG had significantly lower rates of femoral fractures.
Teresi, 2013	Region-based randomization RCT, 1 wk, 12 wks follow-up, 10 sites, CG: 2179 res; IG1: 2255 res; IG2: 2926	Usual care	+	+								IG1: train the trainer sessions for 1–2 high level staff in each NH. Staff training for all frontline staff and additional staff. Basic knowledge about vision and visual impairment and possible interventions for NHs. IG2: same as IG1 plus inspectors (surveyors who are employed by state level departments of health) were trained using same materials as staff.			Reduction in falls for IG1 but not IG2 compared to controls. No changes in behavioral symptoms of dementia. Reduction in depression for IG2 but not IG1 compared to controls.

NRCT, non-randomized controlled trial; RCT, randomized controlled trial; CG, control group; CG1, control group 1; CG2, control group 2; IG, intervention group; IG1, intervention group 1; IG2. Intervention group 2; wks, weeks; res, residents; RN, registered nurse; NA, nursing assistant; GP, general practitioner; NH, nursing home

**Table 10:** Quality improvement.

First author, year	Study design, intervention length, number of sites, baseline sample size	Control condition	Education material	Training	Reminders	Audit and feedback	Mentoring or support	Champion/s	Team meetings	Policy/procedure changes	Organizational restructure	Intervention description	Staff direct behavior outcomes	Staff indirect outcomes	Resident outcomes
Rantz, 2001	Cluster, randomized RCT, 52 wks, 87 sites, CG: 32 facilities; IG1: 27 facilities	Usual care		+		+						IG1: Management and nurse training on quality improvement (QI)and how to use their “Show-Me QI Reports”. Quarterly comparative “Show-Me QI Reports”. Quality Indicator manual and monitoring plans for each Minumum Dataset Quality Indicator. Reference list of clinical standards.	Compared to CG, IGs both declined on presence of little or no activity. No differences between groups over time on use of 9 or more different medications, and prevalence of occasional or frequent bladder or bowel incontinence without a toileting plan, indwelling catheters, daily physical restraints.		No differences between groups over time on incidence of new fractures, and prevalence of falls, behavioral symptoms affecting others, fecal impaction, weight loss, bedfast residents, stage 1–4 pressure ulcers.
	IG2: 28 facilities			+		+	+					IG2: as for IG1 plus telephone and on-site clinical consultation from a gerontological clinical nurse specialist on interpreting reports and deciding about clinical issues that require review.			
Achterberg, 2001	NRCT, 35 wks, 16 sites, CG: 135 res, 16: 143 res	Usual care		+						+		The Resident Assessment Instrument (RAI) structured assessment tool implemented. Project team from each site trained in the RAI method. Trained project teams then trained caregivers in the implementation of RAI.	Assessment: Compared to CG in IG there was significant improvement in taking case history. Management: No differences between groups on care plans, end of shift reports, communication, patient allocation, patient report, total care coordination.		
Crotty, 2004	Paired, cluster randomized RCT, 30 wks, 20 sites, CG: 334 res; IG: 381 res	Usual care	+	+		+	+	+				Academic detailing for physicians on evidence-based guidelines on falls prevention, case audit, stroke prevention. Link nurse for each facility appointed and trained change management, management of behavioral symptoms of dementia, medication management and falls prevention techniques. Staff training on reducing the use of psychotropic medications. All groups received toolkit in managing challenging behaviors.	No difference between groups over time on psychotropic drug use (except for greater use of as required antipsychotics), or on recording of blood pressure, or proportion of patients on aspirin or warfarin.		No difference between groups over time on falls, residents with high blood pressure, or percentage of residents with atrial fibrillation.
Bravo, 2005	Paired cluster randomized RCT, 26 wks, 40 sites, CG: 99 res; IG: 102 res						+			+		Areas for improvement identified and goals developed using goal attainment scaling. Monthly visits to facility and frequent telephone calls to assist manager and staff to implement permanent changes in the areas of care targeted for improvement.	Assessment: Compared to the CG, the IG significantly increased goal attainment scaling. Management: There were no differences between groups on overall quality of care or any sub-dimensions of care.		No differences on overall quality of care between groups. Significant decrease in cognition in the IG over time, but not in the CG.
Baier, 2008	NRCT, 52 wks, 16,756 sites, CG: 9,665 sites; IG: 7,091 sites, N not specified	Usual care					+			+		IG1: Homes set targets using the Nursing Home Setting Targets—Achieving Results (STAR) site for physical restraints and pressure ulcers. IG2: Some homes received additional support from the Nursing Home Quality Improvement Organization.	Compare to CG, IGs had greater improvement for physical restraints. No differences between intervention homes that received or did not receive additional support on physical restraints.		Compared to CG, IGs had greater improvement for pressure ulcers. No differences between intervention homes that received or did not receive additional support on pressure ulcers.
Rantz, 2010	NRCT, 104 wks, 18 sites, CG: 890 res; IG1: 668 res	Usual care		+			+			+		IG1: Implemented Optimus Electronic Medical Record (OEMR) including training. On-site nurse clinical consultation services. Quality Improvement for Missouri (QIMPO) system used.	All groups including controls reduced use of physical restraints. IG1 showed reduction in symptoms of depression with no treatment.	No changes in staff retention over time.	IG1, IG2 and IG3 improved on high risk pressure sores and behavioral symptoms. IG1 and IG2 improved on decline in late-loss activities of daily living, declined in range of motion, declined in urinary tract infections. IG1 and IG3 improved on short stay delirium.
	IG2: 635 res											IG2 –implemented OEMR only including training.			
	IG3: 543 res											IG3 –Used QIMPO only			
Rantz, 2012	Cluster randomized RCT, 104 wks, 58 sites, CG: 29 facilities; IG: 29 facilities; No resident n specified	Videotaped inservices and educational material provided	+				+	+				Detailed intervention manual and text books provided. Researchers observed direct care staff working and provided feedback. Quality improvement teams identified, collected baseline and follow-up data, made and prioritized plans and implemented changes for an area for improvement. Researchers visited monthly to reinforce.	Compared to CG, IG improved on the care subscale of the quality of care measure, but not on the subscales for communication, grooming, environment-access, homelike, odor or environment basics.	No differences between group over time on staff retention, turnover, organizational working conditions, staffing and staff mix.	No differences between groups over time on pressure ulcers, bladder and bowel incontinence, weight loss or decline in activities of daily living.
Van Gaal, 2010, 2011	Cluster randomized RCT, 58 wks, 100 wks follow-up, 10 sites, CG: 127 res; IG: 114 res	Usual care	+	+		+	+	+				Ward manager and 2 key nurses responsible for program implementation. Training for all nurses including education, knowledge test and case discussion. Information collected about pressure ulcers, falls and urinary tract infections. Computerized registration system for documentation of daily care and the presence/absence of adverse events. Feedback given on indicators.	Compared to the CG, the IG residents at risk of falls were more likely to have a preventive plan. No differences between groups on residents at risk of pressure ulcers receiving adequate preventive care. Fewer patients at risk of urinary tract infections in the IG received adequate preventive care.		Compared to CG, IG improved on adverse events (falls, urinary tract infections, pressure ulcers), difference was mainly due to reductions in pressure ulcers and falls.
Boorsma, 2011	Paired, cluster randomized RCT, 26 wks, 10 sites, CG: 139 res; IG: 201 res	Usual care				+			+	+		Resident Assessment Instrument (RAI) used with all residents every 3 months. RAI results compared with benchmarks. Care planning included discussed with resident, family and physician and coordinated by nurse helper. Residents with complex needs scheduled at least twice a year for multidisciplinary meeting. Consultation with a geriatrician or psychologist for frailest residents.	Compared to CG, IG had lower percentage of residents with new in-dwelling catheters, use of physical restraints, use of antipsychotic agents, but higher percentage of residents with inadequate pain management.		No differences between groups on resident rated quality of care, quality of life or activities of daily living, hospital admissions or mortality. Compared to CG, quality of care was higher for the IG using the sum score of 32 risk-adjusted quality care indicators which were mostly resident outcomes.

NRCT, non-randomized controlled trial; RCT, randomized controlled trial; CG, control group; IG, intervention group; IG1, intervention group 1; IG2. Intervention group 2; wks, weeks; res, residents, QI, quality improvement.

**Table 11:** Philosophy of care and aspects of culture of care.

First author, year	Study design, intervention length, number of sites, baseline sample size	Control condition	Education material	Training	Reminders	Audit and feedback	Mentoring or support	Champion/s	Team meetings	Policy/procedure changes	Organizational restructure	Intervention description	Staff direct behavior outcomes	Staff indirect outcomes	Resident outcomes
Burgio, 2001	Paired recruitment, cluster randomized RCT, 5 wks, 8 wks follow-up, 5 sites, CG: 33 res; 25 staff; IG: 34 res, 37 staff	Usual care	+	+		+	+					All nursing staff trained in the use of memory books and general communication skills. Staff motivational system with weekly recognition of staff performance. Observation and feedback to staff on their communication skills.	Compared to CG, over time IG staff improved on overall communication skills, staff positive statements, amount of staff speech and rate of positive verbal interactions between staff and residents during care. There were no differences between groups over time for using fewer multistep instructions, use of biographical statements, or time spent in daily care.		At the 2 month follow-up, IG residents were more independent in self-care than CG.
Schrijnemaekers, 2002, 2002, 2003	Paired, cluster randomized RCT, 35 wks, 52 wks follow-up, 16 sites (8 sites per group), CG: 74 res, 139 staff; IG: 77 res, 154 staff	Usual care, waitlist control		+			+					Clinical lessons for all employees about emotion oriented care. Training for 8 carers per home, supervision meetings.	There were no differences between groups in communication and interaction with the residents.	The IG group had some improvements in job satisfaction and burnout over time compared to the CG.	No intervention effects on behavioral outcome measures over time.
Sloane, 2004; Hoeffer, 2006	Cluster, cross-over RCT (cross over for 2 types of bathing), 6 wks/ 6 wks crossover, 15 sites (5 sites per group), CG: 23 res, 13 staff; IG1: 24 res; IG2: 22 res; IG1 and IG2 combined: 24 staff	Usual care		+		+	+					Three certified nursing assistants per site trained to identify behavioral symptoms and their antecedents, including hands-on supervision to teach: 1) towel bath and 2) person-centered showering. Training on dementia and behavioral symptoms, person-centered approaches to bath, behavioral assessment and problem solving. Review with staff of at least 1 video per resident whom they assisted with bathing.	Bath completeness did not differ from baseline for towel bath or person-centered showering (but person-centered showering took significantly more time than towel bath). Compared to CG, both IG improved more on gentleness and ease, but not verbal support, confidence and hassles.		Compared to CG, both IGs improved in skin condition, and decreased in discomfort ratings, overall agitation and aggression. No differences between two IGs on verbal agitation, or colonization with potentially pathogenic bacteria. Discomfort was less for the towel-bath intervention than the person-centered showering.
Johnson, 2005	NRCT, 5 wks, 17 wks follow-up, 12 sites, CG: 42 res, 18 staff; IG: 42 res, 21 staff	Usual care	+	+								Training for select staff on restorative care: physical activity, positioning, mobility and transfers; communication; feeding/eating; assessment and evaluation. Team building, strategies for motivating residents and decreasing learned helplessness emphasized. Resource manual provided.	Compared to CG, IG improved significantly more in Goal Attainment Scaling (evaluating complex needs of geriatric clients).		Compared to CG, IG improved on functional independence, self-care and progression and recovery in a range of balance and mobility abilities.
Finnema, 2005	Matched then cluster randomized RCT, 39 wks, 16 sites (16 wards from 14 nursing homes), CG: 79 res, 53 staff; IG: 67 res, 46 staff	Training and support in usual nursing home quality care		+			+	+				Basic training in emotion oriented care for all staff and advanced course in emotion-oriented care for five staff members on each ward. One staff member trained as emotion-oriented care advisor who was responsible for implementing emotion-oriented care. Supervision with feedback given.		No differences between groups on general health, perceived work related stress, stress reactions, feelings of competence and number of days to absenteeism due to illness.	Compared to CG, IG had less decline in maintaining a positive self-image and balance among residents with mild-moderate dementia. No differences between groups on resident behavior, depression and agitation.
Berkhout, 2003, 2004; Boumans, 2005	NRCT, 95 wks, 12 sites (12 wards from 3 nursing homes), CG: 109 staff; IG: 101 staff	Usual care					+		+	+	+	Resident-oriented, profession-oriented, and organization-oriented meetings were held. Residents were assigned to primary nursing carers (PNCs), who were responsible for assessment, care planning, execution, and evaluation of care. PNCs were delegated responsibility and accountability for total nursing care of assigned residents. Supervisors coached and support of PNCs. Ward sisters trained on the job and stimulated PNCs.	Compared to CG, IG wards increased more in resident assignment, ‘use of nursing care plans/ evaluation’, ‘taking nursing history’, ‘nursing problems and goals actions’, and (for the psychogeriatric wards only) resident-oriented tasks. IG decreased in quality forms of communication compared to CG. No differences between groups on ward oriented tasks, variety of resident-oriented and ward-oriented forms of communication.	No differences between groups on job autonomy, job responsibility or social support.	No differences between groups on resident wellbeing or resident satisfaction or family satisfaction with care.
Van Weert, 2005, 2006	NRCT, 78 wks, 12 sites, CG: 64 res, 60 staff; IG: 65 res, 60 staff	Usual care	+	+			+			+		CNAs trained in snoezelen for persons with dementia including awareness of residents’ needs; making contact and showing affection and empathy; supporting residents in responsiveness; avoiding correcting the residents’ subjective reality; avoiding spreading useless information and testing knowledge and; in-house supervision offered. Meetings with nursing home representatives to support organizational level implementation.	Compared to CG, over time IG increased time spent in morning care, duration and percentage of eye-contact, affective touch, mean number of smiles, positive affective and instrumental communication, and number of verbal utterances. There was decreased negative affective and instrumental behavior. Increased number of explicitly offered sensory stimuli. Instrumental touch did not differ.	Improvement on Positive Work Scale and Malignant Social Psychology scale.	Compared to CG, the IG improved in duration of resident gaze directed at staff, and frequency of smiling and displayed less disapproval and anger, and more autonomy. There were no differences between groups over time for residents’ positive affective communication, negative instrumental communication or total verbal utterances.
Chenoweth, 2009; Jeon, 2012	Block cluster randomization RCT, 17 wks, 15 sites (3 sites per group), CG: 82 res, 23 staff; IG1: 109 res, 56 staff;	Usual care	+	+			+	+				IG1: two staff from each home trained on person-centered care philosophy and to develop and implement individualized care practices for residents, support visits and phone calls.		Neither intervention resulted in significant lowering of psychotropic drugs. IG2 had decline in the emotional exhaustion, but not depersonalization or person accomplishment, aspects of burnout. Neither intervention resulted in better general health, staff attitudes or reactions to behavioral disturbances, staff perception of support from management.	Compared to CG, over time IG1 and IG2 reduced in agitation. Compared to CG, increase in falls in IG1 and reduction in falls in IG2. No differences between groups on psychiatric symptoms, rate of accidents or hospitalizations.
	IG2: 98 res, 45 staff			+			+					IG2: staff trained in dementia care mapping, peer support groups discussing challenging behaviors, emotional reactions and how to cope with work-related stress. Regular telephone support.			
Burack, 2012, 2012	NRCT, 104 wks, 260 wks follow-up, 13 sites, CG: 51 res; IG: 50 res	waitlist		+				+	+		+	Community coordinators acted as change champions and promoted staffing consistency. Training for all staff on culture change. Staff restructured to work as community teams. Staff to do planned activities with elders. Elders given more choice over their daily schedules. Family invited to be more involved in care planning, community meetings and social events. Environmental changes made.	Compared to CG, overall choice improved for the IG over time. Overall choice for elders increased in the IG compared to CG only from baseline to 2 years, but then decreased from 2 years to 5 year follow-up.		Indirect outcomes: compared to CG, the IG decreased in the frequency of forceful and physical agitation. The frequency of verbal agitation did not significantly decrease.
Clare, 2013	Paired, cluster randomized RCT, 8 wks, 8 sites, CG: 33 staff, 33 res; IG: 32 staff, 32 res	Usual care. Waitlist control		+			+			+		Training on being more aware during care and observational measure of awareness, communication. Staff scheduled to observe a small number of residents. Support offered between weekly training.		No significant differences in staff wellbeing, psychological distress, attitudes or quality of care.	Indirect outcomes: compared to CG, IG significantly improved in family-rated but not staff—rated resident quality of life. No differences on resident well-being, cognitive functioning or behavior.

NRCT, non-randomized controlled trial; RCT, randomized controlled trial; CG, control group; IG, intervention group; IG1, intervention group 1; IG2. Intervention group 2; wks, weeks; res, residents.

**Table 12:** Other studies.

First author, year	Study design, intervention length, number of sites, baseline sample size	Control condition	Education material	Training	Reminders	Audit and feedback	Mentoring or support	Champion/s	Team meetings	Policy/procedure changes	Organizational restructure	Intervention description	Staff direct behavior outcomes	Staff indirect outcomes	Resident outcomes
Molloy, 2000	Paired, cluster randomized RCT, 1 wk, 78 wks, 6 sites, CG: 656 res; IG: 636 res		+	+				+				Training for nurses on advance directives, as health care facilitators, approaches to educating staff, residents and families, and assessing capacity to complete directives. Nurses trained staff and emergency workers. Videos describing program provided. Refresher sessions for new staff and to maintain awareness of already trained staff.	Compared to CG, IG had higher proportion of Advanced Care Directives completed.		Compared to CG, over time IG homes reported fewer hospitalizations per resident and they had a lower mean number of hospital days. Indirect outcomes: differences between groups on satisfaction with health care in competent or incompetent residents or in the proportion of deaths.
Jones, 2004	NRCT, 26 wks, 12 homes, 1899 res total (IG and CG n not specified)	Usual care	+	+		+	+	+		+		Training for all staff, particularly nurses. 3 member internal pain teams (IPTs) formed who developed pain vital sign assessment and documentation. Feedback reports provided. Resident educational video provided on admission. Physicians offered video and pamphlet, CME credits malpractice insurance premium discount. Pain expert available, documentation developed by staff for their facility.	No differences between groups on non-MDS pain assessments and pain reassessments.		No reduction in percentage of residents reporting pain or reporting moderate/ severe pain in IG homes. Improvement in percentage of residents reporting constant pain in intervention homes.
Irvine, 2012	Cluster randomized RCT, 2 wks, 14 wks follow-up, 6 sites, CG: 45 staff; IG: 58 staff	Delayed treatment		+								Internet based training including videos on fundamental de-escalation skills with residents exhibiting aggressive behavior, about hits, hits with fists or arms, hair grabs, wrist grabs.		No differences in self-efficacy or empathy.	Compared to CG, the IG decreased over time in number of assaults reported.
Teresi, 2013	Cluster randomized (region-based) RCT, 4 wks, 52 wks follow-up, 47 sites, CG: 500 staff, 685 res; IG: 525 staff, 720 res	Training in filling out behavior recognition and documentation sheets		+								Staff trained in identification and intervention (and reporting) with respect to resident to resident elder mistreatment.	Compared to CG, IG had higher levels of recognition and documentation of resident to resident mistreatment over time.		
Beeckman, 2013	Cluster randomized RCT, 16 wks, 11 sites, CG: 239 res, 53 staff; IG: 225 res, 65 staff	Pressure ulcer prevention protocol hard copy and lecture for all staff	+	+	+	+		+	+			Electronic decision support system, PrevPlan. Training about pressure ulcer prevention. Monitoring and feedback on adequacy of pressure ulcer prevention, knowledge, attitudes. Reminders. Key nurse introduced, inventory and feedback on quality and availability of current material for pressure ulcers, support the acquisition of new pressure ulcer preventive materials.	Compared to CG, IG more likely to provide fully adequate prevention when in a chair, and in the proportion of residents with some prevention, but no difference between groups over time for fully adequate prevention in bed.		Compared to CG, IG had significantly lower pressure ulcer prevalence (categories I–IV). No difference when only considering categories II-IV.

NRCT, non-randomized controlled trial; RCT, randomized controlled trial; CG, control group; IG, intervention group; IG1, intervention group 1; IG2. Intervention group 2; wks, weeks; res, residents.

## Results

**Fig 2:**
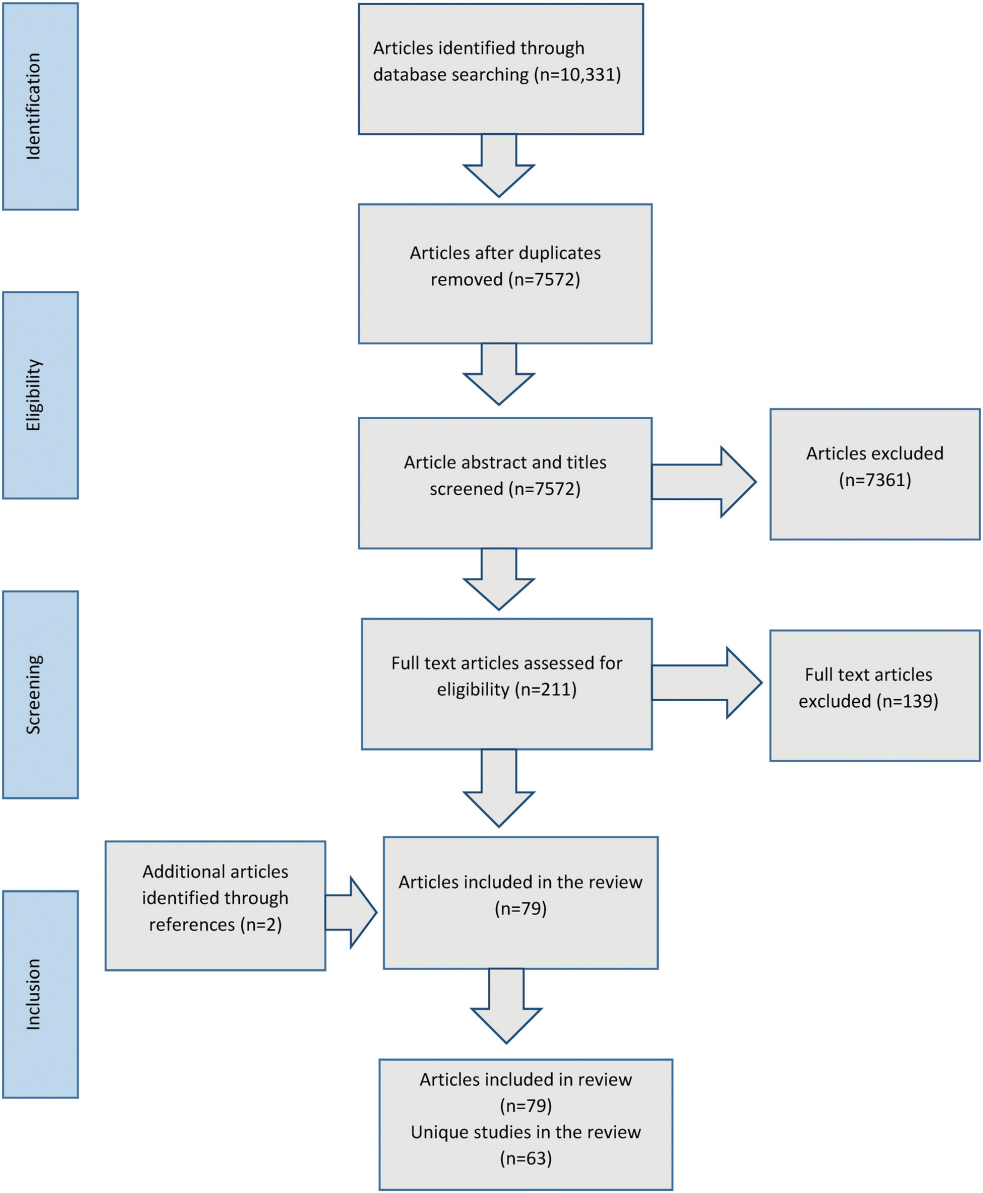
Flow chart indicating inclusion of articles in the study.

The search produced 7572 unique articles, we obtained 211 full text articles and 77 articles were judged to meet inclusion criteria. Two articles were additionally obtained by hand searching reference lists, leading to a total of 79 included articles relating to 63 unique studies. (See Fig 2)

### Oral health

Three studies examined the effect of interventions with staff on the oral health of residents (see Table 1) [28–30]. Two of these were by the same group and tested almost identical interventions in different countries (Belgium and Netherlands) [29,30]. One provided training and toothbrushes [28], the other two provided a more complex multifactorial intervention (De Visschere et al., 2012, van der Putten et al., 2013).

None of the studies measured whether staff behavior changed. All three reported improvements in residents’ denture plaque, and two also improved dental plaque [28,30]. One study also reported improvements on other oral health conditions [28]. See Table 1.

### Hygiene and infection control

Two studies examined the impact of interventions to improve hygiene [31,32] and one focused on infection control [33] (see Table 2). All provided training supported by additional strategies [31–33].

The two studies that reported staff behavior found improvements regarding infection control and hand hygiene. This change in staff behavior resulted in improved outcomes for residents in terms of reducing hospitalization relating to meticillin resistant staphylococcus aureus (MRSA) or respiratory outbreaks [32] but not on prevalence of MRSA [31]. One study reported no impact of the infection control program on resident infections [33]. Staff levels of MRSA were also not shown to be improved in the one study that measured this [31].

### Nutrition

There were two studies that focused on improving residents’ nutrition [34–36] (see Table 3). Both provided training, education materials and supported a change champion. Both studies showed some positive impacts on the nutritional care provided and improvements in some nutritional indicators in residents [35,36] [34].

### Nursing home acquired pneumonia

Two studies used a guideline implementation approach to prevention and management of nursing home acquired pneumonia [37,38] (see Table 4). Both these studies involved staff training, and one supported a nurse to champion the program and materials and reminders [37]. One of the studies also offered staff vaccinations [37]; this showed improvement in staff influenza vaccination rates, and resident pneumococcal vaccination but no differences between groups on antibiotic use [39]. Neither study found differences in the indirect outcomes for residents of hospitalization or short-term mortality [38,40].

### Depression

There were two studies with a focus on reducing nursing home residents’ depression (see Table 5) [41,42]. Both provided staff training. Neither study reported staff outcomes. One study found an the intervention improved depression in somatic but not dementia units and indirect effects of improvement in quality of life for both units [41], however the other study found no impact on depression [42] nor improvements on the more distal resident outcomes of anxiety, quality of life or pain.

### Appropriate prescribing

Seven studies focused on appropriate medication use [43–50](see Table 6), most relating to antipsychotic medications. With the exception of [50], all these studies educated physicians and staff on appropriate medication use and on non-pharmacological strategies to manage clinical conditions. Most studies included other methods in their intervention such as audit and feedback [43,51] and team meetings (i.e. case conferences [44,50,51].

All studies showed improvements in the use of some or all the target medications, and two studies reported no resultant deterioration in resident behavior [44,52]. One study reported both improvements and deteriorations in some resident clinical domains [43]. No studies measured the indirect effects of the programs on staff (i.e. whether they experienced greater stress or perceived workload). Two studies measured effects on indirect resident outcomes and found no effects on falls and wellbeing [52], or on rates of hospitalization, mortality or change in level of care [43].

### Physical restraint reduction

Three studies examined the impact of interventions to reduce physical restraints [53–56] (see Table 7). All included training, and two included consultations [53,56] and one a champion [55].

All these studies measured the impact of training on staff behavior. Two studies reported reductions in physical restraint use by staff without concurrent increase in chemical restraints, or increased falls or injuries in residents [54,55], however one study did not demonstrate overall improvements relative to the control group [53].

### Management of behavioral and psychological symptoms of dementia

There were six studies which attempted to change staff behavior in relation to the management of behavior and psychological symptoms of dementia [57–62](see Table 8). All these involved training and additional intervention components such as mentoring and support [58,59] and reminders [59,62].

Only two of the studies measured whether there was a change in staff behavior in regards to care of people with dementia [58,60]. One study found no changes in the treatment of depression [58] and one improved communication in pain awareness [60]. One study showed that the intervention had negative impacts on staff stress and perception of supervisory support [60]. However, another study that measured indirect staff outcomes and found benefits for emotional reactions, autonomy and work pleasure, but not for the more distal outcomes of general health and job satisfaction [61]. The effects of the interventions on resident behavior were mixed, with some studies suggesting behavioral improvements [58,59], some studies finding worsening of behaviors [61,62]. One study which measured the indirect resident outcome of quality of life found no impact [61].

### Falls reduction and prevention

Eleven studies examined interventions to change staff care practices with regards to falls reduction and prevention [63–74] (see Table 9). Compared with other clinical domains the studies in this domain tended to be larger in terms of number of sites and participants. With one exception which introduced a computerized measure of fall reporting [67], all the studies offered training and additional intervention components such as education materials [63,65,66,72,74] hip protectors [63,74], reminder materials [63,70], assessment tools [64,68,71], supported behavior change [66,68], audit and feedback [64,71], champions [64,71,72], and one trained regulatory inspectors [73]. Two studies also encouraged staff to run exercise groups [71,72].

Five of the studies examined whether staff changed their practices though none examined fall prevention activities comprehensively—one reported increases in hips protector use [65] and two did not [63,69], one found some improvement in documentation relating to falls [67], one reported reductions in physical restraint use and better care process documentation for falls [68], and one found increased prescriptions of biphosphonate, calcium and Vitamin D [69]. Of the seven studies which investigated the impact of the intervention on rates of falls [64,65,67–70] three reported a reduction [64,70,73]. Only one of eight studies which looked at rates of fractures or other injuries [63–66,68,69,71,72] found a reduction in at least one type of injury [72]. One study found a reduction in hospitalization related to falls [65].

### Quality improvement

Nine studies investigated the impact of interventions to improve care quality [75–83] (see Table 10). With one exception [79], all studies utilized multi-component interventions which encouraged nursing homes to examine their existing care performance and processes and included other methods to support facilities to change their practices.

All studies included and reported improvements in at least one measure of staff behavior related to quality improvement [75–77,79–82,84,85]. The studies which measured the impact of quality improvement on indirect staff outcomes such as retention rates found no effect [82,83]. Five studies which measured outcomes for residents reported improvements on at least one of these [78,79,81,83,85]. The two which did not improve resident outcomes produced minimal changes in staff behavior [76,77]. There did not appear to be a pattern in type or number of components of interventions and outcomes.

### Philosophy of care

Ten studies focused on changing the philosophy or aspects of care culture, such as person-centered care, emotion-oriented care, awareness oriented care and restorative care [86–103](see Table 11). One study offered training only [91], and one changed staff responsibilities and care procedures [93]. The remaining studies combined training with other intervention components such as mentoring or support [86,87,89,92,97,99,100,102] and audit and feedback [86,89].

Seven out of eight studies that measured whether staff changed their behavior showed at least some improvements [86,90,91,94,96,98,101]. There did not appear to be a pattern in the intervention components that produced successful interventions. Studies that measured indirect staff outcomes reported improvements relating to feelings related to some aspects of work [88,96,101,102], but not on more distal outcomes of health, stress, absenteeism or turnover [92,93,100,101]. Some studies reported benefits on resident behavior [87,89,97,98] functional ability and self-care [91] and quality of life [100], however others found no change or a negative effect on behavior [92,100], wellbeing and satisfaction with care [95,100] and resident communication [102]. Generally, studies which had positive outcomes for residents also achieved staff care practice change, however changing staff behavior did not necessitate improved resident outcomes.

### Other clinical domains

Single studies were identified which addressed use of advance care directives [104], pain management [30], assault reduction [105], resident to resident mistreatment [106], and pressure ulcer reduction [107](see Table 12). Three of these studies had some positive effects for changing staff practices [104,106,107] and the three studies which reported resident outcomes showed some positive results [104,105,107].

### Theoretical orientation of practice change component of program

Nine of the eleven studies that reported using a theory in planning the intervention successfully changed at least one aspect of staff care practices. The theories were: Kotter’s eight-step change model [82], Kitson implementation of evidence based practice framework [35,36,63], precede/proceed model [60], Roger’s diffusion theory of innovation [37,39,40,108], Bandura’s social learning theory [65,74], adult learning theory [106], Grol and Wensing’s stepwise approach to implementation [107], the disease management model [81], and the theory of planned behavior [55].

### Program logic

Only three studies explicitly described or presented their program logic or how the intervention was intended to impact the outcomes measured. Zimmerman [60] provided a clear causal chain (and means of testing it) from staff training through to resident quality of life. Teresi [73] provided a risk factor model indicating risk factors, process outcomes and distal outcomes. Smith [42] outlined the components of nursing home staff participation and resident participation in the program and the evaluation methods for each level of participation.

The program logic models that we drew based on the intervention description show that targeted staff practices were often not evaluated (for example in Fig 1 in the Meyer study, staff falls prevention practices were not measured), or only some aspects of practice change were evaluated. The logical link was not always apparent or strong between the intervention elements and some of the indirect staff outcomes, particularly turnover and absenteeism, and resident outcomes such as quality of life.

### Translating research-demonstrated programs

Three studies reported implementing with staff a program which had previously been shown to be effective when delivered by expert clinicians [38,61,66]. These were in the areas of NHAP guideline adherence, fall-related injury prevention and dementia care. There were also two studies which were larger implementation projects of a fracture prevention program which was originally shown to be effectively delivered by staff [71,72,109].

### Potential barriers and enablers to change

Some studies reported barriers and enablers as part of a formal process evaluation [e.g.s 54,105,110] and others reported barriers as part of the discussion [e.g.s 53,92,107]. Many barriers and enablers related to staff—these appeared to be factors that impact on staff practices in general as well as in the implementation of new practices (e.g. high turnover, absenteeism, high workload, low education, and communication/support from senior staff). Organizational and system issues cited seemed to be more specific to the implementation of the new practices e.g. insufficient funding, logistical issues and infrastructure difficulties associated with implementation. Finally, there were several studies that mentioned barriers and enablers that were related to the resident’s high care needs or attitudes of residents and/or families (see Table 13).

**Table 13:** Barriers and enablers to change.

	Barrier	First author, year
Staff		
	High turnover or absenteeism	Hutt, 2010; Kerse, 2004; Wagner, 2005; Rask, 2007; Rantz, 2001; Achterberg, 2001; Crotty, 2004; Rantz, 2012; Teresi, 2013a; Bravo, 2005; Teresi 2013b.
	High workload	Ho, 2012; Rantz, 2001; Schrijnemaekers, 2003; Johnson, 2005; Boumans, 2005; Teresi 2013a; Bravo, 2005; Teresi, 2013b; van Weert, 2004.
	Insufficient support from senior staff	Kerse, 2004; Boumans, 2005; van Weert, 2004; Huizing, 2009.
	Opposing attitudes and lack of commitment	De Visschere, 2012; Rask, 2007; Rantz, 2001; Baier, 2008.
	Low education	Ho, 2012.
	Not all staff trained in intervention	Leone, 2013; Crotty, 2004; Huizing, 2009.
	Communication/ cooperation between staff/ physicians	Kerse, 2004; Becker, 2011; Johnson, 2005; van Weert, 2004.
Organisation/ systems issues		
	Funding and resources lacking	Ho, 2012; Avorn, 1992; Ray, 2005; Rask, 2007; Johnson, 2005; Beeckman, 2013.
	Infrastructure/ software difficulties	Wagner, 2005; Achterberg, 2001; Irvine, 2012.
	Difficulties with logistics (e.g. time schedules, organization)	Crotty, 2004; Zimmerman, 2010; Schrijnemaekers, 2003; Berkhout, 2003.
	Does not align with other guidelines/ framework/ policies	Avorn, 1992; Ray, 2005; Schrijnemaekers, 2003.
	Competing priorities	Teresi, 2013b; van Weert, 2004.
	Traditional culture	Fossey, 2006; Berkhout, 2003; Irvine, 2012.
Resident/ family		
	Residents’ high level of care needs	Stein, 2001; Eisses, 2005; Kerse, 2004; Boumans, 2005.
	Resident/ family attitudes	De Visschere, 2012; Hutt, 2010; Schrijnemaekers, 2003.
Other		
	Complexity in establishing best practice	Crotty, 2004.
	Insufficient length of intervention	Finnema, 2005.
	External opinion leaders	Hutt, 2011; Gulpers, 2013; Becker, 2011.

### Risk of bias (see data in S1 Appendix and Tables 1–12)

Given the nature of staff behavior change interventions, the allocation to control and intervention groups were not blinded from almost all staff participant groups. Some studies did not use a randomized design, and randomized trials often did not report on their randomization method. Other common biases were incomplete outcome data not being adequately addressed and assessors not being blinded to group allocation. Most studies took some care to protect against contamination and we were unable to detect selective outcome reporting. Baseline characteristics and outcomes were usually similar or controlled for to the intervention group. However, studies which were rated as having multiple risks of bias were not more likely to report a positive outcome.

## Discussion

There are no “magic bullets” to change staff care practices in order to improve resident outcomes. We did not find that any single intervention component (e.g. champions, or audit and feedback), or combination of components consistently resulted in improvements in staff practices within each clinical domain, nor did increasing the number of intervention components.

Studies that did not change the targeted staff behavior tended to also not improve resident outcomes, and indirect staff outcomes were rarely improved as a result of interventions aimed at improving care of residents.

Studies in clinical domains involving more specific care practices (i.e. hygiene, oral care, appropriate prescribing, and physical restraint reduction) tended to have a higher proportion of “successful” studies compared to domains requiring more global practice changes (i.e. dementia care, falls, quality improvement, philosophy of care). Possible reasons for these differences are:

- The staff behaviors were relatively easier to target and easier to change, such as those which require the changes during specific care practices by individual staff, rather than more coordinated changes between staff across multiple care practices
- The target outcomes were easier to measure (and therefore successes and failures were easier to observe)
- The primary outcome of the intervention was staff behavior which is more directly influenced by the intervention components, rather than resident outcomes
- There was a better established evidence base between specific care practices and resident outcomes (e.g. fracture prevention program by [109], or between implementation strategies and changing that behavior in another setting (e.g. hygiene in hospitals [111])

In many studies the logical relationships between interventions and measured staff and resident outcomes were not clear. Using a program logic model may help better match intervention components and outcomes in designing the intervention and measurements, as well as assisting with maintaining program integrity during delivery. The program logic model also can guide choice of outcome measures, measuring resident outcomes address questions of effectiveness, and may help researchers and services wanting replicate the intervention in their own setting understand the how practice changes were achieved [112]. When staff behavior is not measured, it is not clear whether the program has been unsuccessful because of implementation error or because the staff behavior has changed, but has not brought about the desired improvement in residents [113].

These results support the notion that using theory to plan implementation strategies will increase the success of translating research into practice change [114]. Theories are seldom used, possibly because of the proliferation of theories, models and frameworks, many with limited empirical validation [115]. Nilsen has suggested that since implementation is multifaceted and complex it is unlikely that a single theory can guide all endeavors in the field, however those of us attempting to change practice are left with little guidance on how to choose a theory to guide our implementation.

Barriers and enablers for staff behavior change were often discussed in the context of failed or suboptimal interventions; addressing these proactively as part of the intervention design may increase the chances of success [116]. Common barriers at the staff level were high turnover or absenteeism and high workload, and at the organizational level, were lack of resources and funding, infrastructure and software difficulties and other logistic difficulties such as time scheduling and organization. Barriers were consistent with other research relating to practice change in nursing homes [117,118]. Researchers should consider barriers from staff, organizational and resident and family perspectives, as well as the external context.

### Strengths and limitations

The inclusion criteria were designed to include higher quality studies; however we may have inadvertently missed a high quality study because of how we operationalized inclusion criteria. There may be a risk of publication bias towards reporting of studies with positive effects within the literature and also of selective reporting within studies—we were unable to assess publication bias statistically as the range of outcomes within each group of studies meant that it did not make sense to combine them in a forest plot. The patterns of results described in this review should be considered with this limitation in mind.

We included a broad range of staff behavior changes so that we could observe the common ingredients for successful practice change across interventions. This was challenging because of the large number of studies included. We decided to present the studies grouped according to clinical domains, we examined groupings according to intervention components, however these were difficult to interpret and not very meaningful. The review team ranked difficulty and complexity of behavior change of clinical domains subjectively.

This review did not look at the ‘dose’ of training or other components, just whether these were provided. This was not examined because the length and frequency of training were usually described however other aspects such as the number of staff trained and style of training (e.g. didactic, interactive) were not routinely reported and may also be important in influencing the impact of training. We attempted to examine fidelity of implementation of the interventions, however this was poorly described or not described at all in many studies, such that is it not known whether the interventions were delivered as described in many studies.

### Practice change and research implications

Researchers, clinicians and service providers contemplating programs requiring staff behavior change in nursing homes should consider: a multifactorial program rather than training alone, investigating and addressing barriers and enablers for their program, using a theory and program logic to design the intervention to ensure that components that target the specific behaviors they want to change and considering motivation as well as knowledge and skills, conducting a process evaluation based on the theory and program logic so as to understand how and why the program succeeds or fails, and planning their statistical analyses to take into account clustering and incomplete datasets.

Future research could consider staff motivations in achieving and sustaining behavior change, distinct from delivery of the knowledge and skills required for the change. It would also be useful to develop of a list of common barriers and possible solutions in nursing home practice change, as well as a framework for categorizing the difficulty or complexity of behavior change, for individuals and in an organizational context. The methodology for systematic reviews of efficacy (examining the relationship between a single intervention and single outcome) is well developed, similar methodological development is required for systematic reviews of complex interventions [119] and when outcomes relate to implementation success.

## Supporting Information

S1 Appendix Risk of bias ratings. (PDF)

S1 PRISMA Checklist PRISMA Checklist. (PDF)
