# Supplementary material for: A Systematic Review of Interventions to Change Staff Care Practices in Order to Improve Resident Outcomes in Nursing Homes
Source: PLoS One. 2015 Nov 11;10(11):e0140711. doi: 10.1371/journal.pone.0140711 (PMC4641718; doi:10.1371/journal.pone.0140711)
Supplement: S1 Appendix — (PDF) [file pone.0140711.s001.pdf]

S1 APPENDIX

Risk of bias ratings

First author	Year	Title	Bias_no random allocation	Bias_allocation not concealed	Bias_baseline outcomes not similar	Bias_baseline characteristics not similar	Bias_incomplete data not addressed	Bias_not blindly assessed	Bias_contamination not prevented	Bias_selective outcome reporting	Bias_other biases	Bias_clustering not analyzed
Achterberg	2001	Improvements in the quality of co-ordination of nursing care following implementation of the Resident Assessment Instrument in Dutch nursing homes	X	X	X		U	X	U			
Avorn	1992	A randomized trial of a program to reduce the use of psychoactive drugs in nursing homes	U	X			U					
Baier	2008	Aiming for star performance: the relationship between setting targets and improved nursing home quality of care	X	X	X		U	X	X		X	
Baldwin	2010	Cluster randomized controlled trial of an infection control education and training intervention programme focusing on meticillin-resistant Staphylococcus aureus in nursing homes for older people		X			U	X				
Becker	2011	Reduction of femoral fractures in long-term care facilities: the Bavarian fracture prevention study.	X	X			U		X	X	X	X
Beeckman	2013	A multi-faceted tailored strategy to implement an electronic clinical decision support system for pressure ulcer prevention in nursing homes: a two-armed randomized controlled trial		X			U	U				

Berkhout; Berkhout; Boumans	2003, 2004; 2005	Effects of resident-oriented care on job characteristics of nursing caregivers	X	X	X		U	X	U			X
Boorsma	2011	Effects of multidisciplinary integrated care on quality of care in residential care facilities for elderly people: a cluster randomized trial.		X	U				U			
Boumans	2005	Effects of resident-oriented care on quality of care, wellbeing and satisfaction with care	X	X				U	X			
Bouwen	2008	Rate of accidental falls in institutionalized older people with and without cognitive impairment halved as a result of a staff-oriented intervention	U	X	U		X	X				X
Bravo	2005	Using goal attainment scaling to improve the quality of long-term care: a group-randomized trial	U	X								
Burack; Burack	2012; 2012	The impact of culture change on elders' behavioral symptoms: a longitudinal study	X	X	U	U	X	X	X	U	U	X
Burgio	2001	Come talk with me: improving communication between nursing assistants and nursing home residents during care routines	U	X			X	X				X
Chenoweth	2009	Caring for aged dementia care resident study (CADRES) of person-centered care, dementia-care mapping, and usual care in dementia: a cluster randomized trial		X								
Clare	2013	AwareCare: a pilot randomized controlled trial of an awareness-based staff training intervention to improve quality of life for residents with severe dementia in long-term care settings	X	X	X		U					X
Cox	2008	Educating nursing home staff on fracture prevention: a cluster randomized trial		X	U		U					

Crotty	2004	An outreach intervention to implement evidence based practice in residential care: a randomized controlled trial		X			X			X		
Crotty	2004	An outreach geriatric medication advisory service in residential aged care: a randomized controlled trial of case conferencing		X				U	X	X	U	X
De Visschere	2010	Effect evaluation of a supervised versus non-supervised implementation of an oral health care guideline in nursing homes: a cluster randomized controlled clinical trial.	U	X								
Deudon	2009	Non-pharmacological management of behavioral symptoms in nursing homes	U	X	X							X
Eisses	2005	Care staff training in detection of depression in residential homes for the elderly: randomized trial	U	X								
Finnema	2005	The effect of integrated emotion-oriented care versus usual care on elderly persons with dementia in the nursing home and on nursing assistants: a randomized clinical trial.	U	X				X			U	
Fossey	2006	Effect of enhanced psychosocial care on antipsychotic use in nursing home residents with severe dementia: cluster randomized trial.		X			U					
Frenkel	2001	Improving oral health in institutionalized elderly people by educating caregivers: a randomized controlled trial		X								
Gaskill	2009	Maintaining nutrition in aged care residents with a train-the-trainer intervention and nutrition coordinator			U	U	X	U				
Gulpers; Gulpers	2011; 2013	Belt restraint reduction in nursing homes: effects of a multi-component intervention program	X	X			X					U

Ho	2012	Effectiveness of multifaceted hand hygiene interventions in long-term care facilities in Hong Kong: a cluster randomized controlled trial.		X		X		X				
Hoeffer; Sloane,	2006; 2004	Assisting cognitively impaired nursing home residents with bathing: effects of two bathing interventions on caregiving.	U	X			X					
Huizing	2009	A cluster randomized trial of an educational intervention to reduce the use of physical restraints with psychogeriatric nursing home residents.	U	X			X		X			X
Hutt; Hutt; Linnebur	2011, 2009, 2011	A multifaceted intervention to implement guidelines did not affect hospitalization rates for nursing home-acquired pneumonia	X	X			U	X				X
Irvine	2012	An internet training to reduce assaults in long-term care	U	X			X	X				
Johnson	2005	Evaluation of the restorative care education and training program for nursing homes	X	X			X					X
Jones	2004	Translation research in long-term care: Improving pain management in nursing homes	X	X	U	U	X	U			X	
Kerse	2004	Fall prevention in residential care: a cluster, randomized, controlled trial		X								
Kopke	2012	Effect of a guideline-based multi-component intervention on use of physical restraints in nursing homes: a randomized controlled trial		X			U					
Leone	2013	Management of apathy in nursing homes using a teaching program for care staff: the STIM-EHPAD study	U	X						U	U	X
Leontjevas	2013	A structural multidisciplinary approach to depression management in nursing-home residents: a multicentre, stepped-wedge cluster-randomized trial.		X								

Makris	2000	Effect of a comprehensive infection control program on the incidence of infections in long-term care facilities	U	X	X	X		X				
Meador	1997	Predictors of antipsychotic withdrawal or dose reduction in a randomized controlled trial of provider education	U	X			X					X
Meyer	2003	Effect on hip fractures of increased use of hip protectors in nursing homes: cluster randomized controlled trial		X								
Molloy	2000	Systematic implementation of an advance directive program in nursing homes	U	X			U	U				
Naughton	2001	Antibiotic use, hospital admissions, and mortality before and after implementing guidelines for nursing home-acquired pneumonia	U									X
O'Halloran	2004	A cluster randomized controlled trial to evaluate a policy of making hip protectors available to residents of nursing homes	U	X	U	U	X	X				
Proctor; Proctor	1998, 1999	Behavioral management in nursing and residential homes: a randomized controlled trial.		X	X		U	U				
Rantz	2012	Randomized multilevel intervention to improve outcomes of residents in nursing homes in need of improvement.	U	X			X	X				X
Rantz	2001	Randomized clinical trial of a quality improvement intervention in nursing homes.	U	X			X	X				
Rantz	2010	Cost, staffing and quality impact of bedside electronic medical record (EMR) in nursing homes	X	X	U		X					X
Rapp	2010	Effect of a statewide fall prevention program on incidence of femoral fractures in residents of long-term care facilities		X	U		U	X	X			U
Rask	2007	Implementation and evaluation of a nursing home fall management program	X	X				X	X			

Ray	2005	Prevention of fall-related injuries in long-term care		X	U			U				
Schmidt	1998	The impact of regular multidisciplinary team interventions on psychotropic prescribing in Swedish nursing homes	U	X			X	U				X
Schrijnemaekers; Schrijnemaekers	2002a, 2003	Effects of emotion-oriented care on elderly people with cognitive impairment and behavioral problems.	U	X				X				
Smith	2013	Depression training in nursing homes: lessons learned from a pilot study	X	X			X	X				X
Stein	2001	Educational program for nursing home physicians and staff to reduce use of non-steroidal anti-inflammatory drugs among nursing home residents.	U	X			X					
Teresi	2013	Comparative effectiveness of implementing evidence-based education and best practices in nursing homes: Effects on falls, quality-of-life and societal costs			X			X				
Teresi	2013	A staff intervention targeting resident-to-resident elder mistreatment (R-REM) in long-term care increased staff knowledge, recognition and reporting: results from a cluster randomized trial		X					X			
van de Ven	2013	Effects of dementia-care mapping on residents and staff of care homes: a pragmatic cluster-randomized controlled trial.		X				U				
van der Putten	2012	Effectiveness of supervised implementation of an oral health care guideline in care homes; a single-blinded cluster randomized controlled trial	U	X								

van Gaal	2011, 2011	Fewer adverse events as a result of the SAFE or SORRY? Programme in hospitals and nursing homes. Part I: Primary outcome of a cluster randomized trial		X				U				
van Weert ; van Weert	2004, 2006	Effects of snoezelen, in 24 h dementia care, on nurse-patient communication during morning care	X	X					X			X
Wagner	2005	Impact of a falls menu-driven incident-reporting system on documentation and quality improvement in nursing homes.	X	X	U	U		X		U	X	X
Westbury; Westbury	2010, 2011	An effective approach to decrease antipsychotic and benzodiazepine use in nursing homes: the RedUSe project.	X	X				X				
Westergren; Westergren ,	2009,2010	Do study circles and a nutritional care policy improve nutritional care in a short- and long-term perspective in special accommodations?	X	X		U	X	X		U		X
Zimmerman	2010	Outcomes of a dementia care training program for staff in nursing homes and residential care/assisted living settings	U	X		U		U				

X – presence of bias, U – unclear whether bias is present
